# New Molecules and Old Drugs as Emerging Approaches to Selectively Target Human Glioblastoma Cancer Stem Cells

**DOI:** 10.1155/2014/126586

**Published:** 2014-01-02

**Authors:** Roberto Würth, Federica Barbieri, Tullio Florio

**Affiliations:** Section of Pharmacology, Department of Internal Medicine and Centre of Excellence for Biomedical Research (CEBR), University of Genova, Viale Benedetto XV, 2 16132 Genova, Italy

## Abstract

Despite relevant progress obtained by multimodal treatment, glioblastoma (GBM), the most aggressive primary brain tumor, is still incurable. The most encouraging advancement of GBM drug research derives from the identification of cancer stem cells (CSCs), since these cells appear to represent the determinants of resistance to current standard therapies. The goal of most ongoing studies is to identify drugs able to affect CSCs biology, either inducing selective toxicity or differentiating this tumor cell population into nontumorigenic cells. Moreover, the therapeutic approach for GBM could be improved interfering with chemo- or radioresistance mechanisms, microenvironment signals, and the neoangiogenic process. During the last years, molecular targeted compounds such as sorafenib and old drugs, like metformin, displayed interesting efficacy in preclinical studies towards several tumors, including GBM, preferentially affecting CSC viability. In this review, the latest experimental results, controversies, and prospective application concerning these promising anticancer drugs will be discussed.

## 1. Glioblastoma

Glioblastoma (GBM), classified by World Health Organization as grade IV astrocytoma, is the most common primary brain tumor in adults, accounting for more than 50% of all gliomas. Main features responsible for GBM aggressiveness are high cell proliferation rate, diffuse infiltration within brain parenchyma, marked angiogenesis, and genomic instability, all of them resulting in drug resistance. As a consequence, GBM patients follow a rapidly lethal clinical course. Presently, the therapeutic strategy to improve the survival of GBM patients is based on a multimodal approach which includes maximal cytoreductive surgery followed by a combination of radiation and adjuvant chemotherapy with temozolomide (TMZ) [1].

Besides establishing a definitive histopathological diagnosis, surgery, according to tumor location, size, and growth rate, and to the age of the patient, leads to rapid improvement of clinical symptoms. If nearly total resection is feasible, it may facilitate adjuvant therapy. However, due to the infiltrating behavior of GBM cells, the complete resection is generally unachievable. Indeed, surgery is usually able to remove over 90% of the tumor bulk, but microscopic total resection is not possible since GBM cells migrate away from the tumor mass and lead to relapse. Thus, prognosis of GBMs is poor, although extensive surgery enhances the quality of life of patients reducing mass effects and is associated with a slightly longer life expectancy [2]. Postsurgery therapy consists of focal radiotherapy (RT) at the primary tumor site. Stereotactic radiosurgery or fractionated stereotactic irradiation represents alternative approaches for relapsing tumors. Although RT prolongs survival of GBM patients compared to surgery alone, the responsiveness of GBM to RT is extremely variable, inducing a transitory phase of remission, characterized by stability or regression of neurologic deficits as well as reduction of the tumor size, followed by tumor recurrence, resulting in further fatal clinical decline within one year [3].

Survival benefits have been also obtained with the introduction of the oral alkylating agent TMZ, as RT-concurrent or adjuvant therapy [4]. TMZ is a small (194 Da), lipophilic prodrug that, at physiologic pH, is rapidly converted into the active metabolite 3-methyl-(triazen-1-yl)imidazole-4-carboxamide (MTIC) by nonenzymatic chemical degradation process. MTIC prevents cell division by interrupting normal DNA replication.

Unfortunately, long term survivors are rare, and the frequent recurrence after TMZ therapy highlights the presence of TMZ-resistant GBM cells. Indeed, resistance to TMZ is mainly mediated by high level of the DNA repair enzyme O6-methylguanine methyltransferase (MGMT), which repairs TMZ-induced DNA adducts. So far, MGMT seems to be one of the most significant mechanisms of chemoresistance in GBM [5].

Furthermore, MGMT promoter methylation is associated with a significantly higher median survival after therapy with TMZ [6], and MGMT methylation status is a helpful predictive biomarker for the response to TMZ or other alkylating agents [7].

Despite the above described aggressive multimodal standard of care, median overall survival is just 14.6 months, as compared to 12.1 months with radiation alone [4]. Thus, being GBM still almost incurable, the requirement of new drugs is a compelling requirement.

Presently, preclinical and clinical studies are focused on (I) the identification of mechanisms to overcome TMZ resistance, (II) the development of molecular targeted and antiangiogenic agents, (III) immunotherapy, and (IV) drug combination.

Many chemotherapeutic agents and new molecular targeted compounds have been tested and are currently under investigation in clinical trials. So far, however, only bevacizumab, a humanized monoclonal antibody against vascular endothelial growth factor (VEGF), was approved by the U.S. FDA in 2009 as a single agent for recurrent GBM [8], although a significant impact on overall survival was not observed [9].

## 2. Cancer Stem Cells as New Pharmacological Target for GBM

Recent studies showed that, like normal tissues, GBM is composed of heterogeneous cell populations, concerning morphological and differentiation status [10]. It was proposed that a small population of tumor cells, named cancer stem cells (CSCs) due to normal stem-cell-like features, is responsible for origin, growth, recurrence, and drug resistance of several blood and solid tumors, including GBM. While nowadays largely accepted, CSCs hypothesis still remains a subject of discussion and controversy. In the last years this theory has greatly evolved in order to compose some incongruences regarding hallmarks of this cancer cell population, and in particular concerning the plasticity of CSCs [11]. The main reason that ignites this debate is the variability of the experimental conditions adopted in the different studies (i.e., *in vitro*/*in vivo*/*ex vivo* models), which affects the detection and frequency of CSCs within solid tumors, and whether stemness of CSCs is a phenotypic property of some cancer cells at a certain time rather than a defined cell subpopulation [12–15]. Although all the different CSC features did not necessarily exist simultaneously in the same cells, some functional and biochemical features are recognized as stemness hallmarks and are beginning to be exploited to develop therapeutic regimens that can prevent the emergence of tumor stem-like cell variants able to drive tumor formation.

Unique features of CSCs, making them a relevant key in tumor survival, are as follows.

1. Self-renewal: the process by which CSCs divide with the maintenance of the undifferentiated state, sustaining the CSC pool within the tumor mass. By asymmetric division, every CSC generates an identical daughter cell and a progenitor cell able to differentiate and proliferate but with reduced ability to self-renew.
2. Multilineage differentiation: the capacity to give rise to heterogeneous populations of cancer cells that constitute the tumor, leading to a hierarchy of cells within the neoplasia.
3. Tumorigenicity: CSCs are the only cancer cells able to initiate and recapitulate the original malignancy when xenotransplanted in animal models (i.e., immunocompromised mice), so that they are also called tumor-initiating cells (TICs) in order to highlight their tumorigenic potential. Moreover, CSCs are much more chemo- and radioresistant than differentiated cells forming the tumor mass, and thus they are believed to be responsible for drug resistance and tumor recurrence [16, 17].

Notably, besides the above mentioned properties (self-renewal potential and ability of multilineage differentiation), GBM-derived CSCs share a crucial feature with neural stem cells: the expression of distinctive stem/precursor markers that can be helpful to discern them from non-CSC populations within a tumor. Indeed, one of the most used *in vitro* or *ex vivo* method to isolate and recognize CSCs within a tumor is the phenotypic characterization.

Among all the markers identified so far, the five-transmembrane domains glycoprotein CD133, also called prominin 1, seems to be the most reliable candidate surface marker for GBM CSC. Human CD133^+^ GBM stem cells are able to recapitulate the original tumor when injected into brain of immunodeficient mice [18] and it was recently demonstrated, both *in vitro *and *in vivo,* that CD133 is essential for self-renewal and tumorigenic potential of GBM stem/progenitor cells [19]. Moreover, CD133 expression levels have been correlated with adverse GBM clinical outcome in a number of studies [20]. In light of this evidence, CD133 is often considered the chief marker for the identification of GBM CSCs. However, several studies reported that tumorigenic activity, the main operational definition of CSCs, also occurs in CD133-negative GBM cells, and the reliability of CD133 for the isolation of brain tumor stem cells is highly questioned [21]. Additionally, CD133 is not uniformly expressed by GBM CSCs themselves, owing to population heterogeneity.

Due to the lack of absolute criteria and uniform biomarkers, in literature different methods for the isolation/enrichment of CSCs are reported. Thus, the phenotypic characterization needs to be validated with functional properties to establish the presence of CSC in *in vitro* cultures.

Considering all these aspects, the nomenclature of these cells has been actually controversial. To avoid further variability, in this review we adopt interchangeably tumor-initiating cell (TIC) and cancer stem cell (CSC) terms emphasizing the functional meaning.

GBM CSC origin is still unclear and debated. CSCs could derive either from normal neural stem or progenitor cells that have accumulated mutations as a result of intrinsic events, such as sequential genetic or epigenetic mutations, and/or extrinsic events mediated by the microenvironment. It has also been hypothesized that CSCs arise from spontaneous dedifferentiation of tumor cells or dynamic interchange between CSCs and progenitors cells [22]. Another intriguing possibility is that differentiated neurons and/or astrocytes can be transformed and dedifferentiated by oncogenes and originate tumors that recapitulate the cell heterogeneity of human GBM [23].

Since GBM therapy failure is mainly due to tumor recurrence, in which CSCs are thought to have a key role, the identification of CSC in GBM has led to a stimulating innovation of modern drug investigation [24].

Indeed, anticancer research challenge for the next years is the developing of strategies able to target this cell subpopulation to succeed in affecting GBM outcome.

Interestingly, the effects of chemotherapeutic drugs, such as TMZ, on GBM CSCs viability are still a controversial topic [25]. Indeed, a significant GBM CSC resistance towards TMZ was observed in some studies [26–28], while others showed a dose- and time-dependent susceptibility of CSCs to TMZ exposure [29]. This variability, likely due to the different experimental settings, in particular considering the definition of CSCs, highlights the requirement of additional data to obtain concordant results and the development of consensus protocols to assess CSCs biological properties in preclinical studies. However, the different sensitivity of CSCs towards TMZ can be at least in part explained by the variability in MGMT expression in the original tumors from which CSCs were isolated [30].

The above described complexity and controversy of drug resistance of an evolving population of cells as CSCs suggest that simultaneous multitarget therapy represents the only approach able to avoid cells, already resistant to one drug, to survive and acquire later resistance to other compounds.

Potential direct approaches to eradicate CSCs are aimed to interfere with pathways involved in the maintenance of the fundamental characteristics of these cells: self-renewal and proliferation, surface markers, chemoradiation resistance, or inducing differentiation.

Other strategies to indirectly inhibit or differentiate CSCs are aimed to deregulate the microenvironment where CSCs and progenitors reside [24].

GBM tissues show high vascular endothelial proliferation and large necrosis areas, possibly related to hypoxic microenvironment that regulates CSCs behavior and recruitment of vascular and stromal cells able to promote angiogenesis and tumor growth [31]. The interaction between GBM CSCs and different cell types that reside in the microenvironment may modulate CSC invasiveness and intrinsic drug resistance. GBM cells highly express VEGF and the CXCL12/CXCR4 chemokinergic system [32–34] that act as proangiogenic and migratory factors and likely contribute to feeding self-renewal of CSCs. Thus, chemokine CXCL12 through the autocrine/paracrine activation of its receptor, CXCR4, also proposed as surface marker for GBM CSCs [35], may represent a valuable target to block GBM CSCs self-renewal [36], or their invasive behavior [37], using CXCR4 antagonists such as the clinically approved drug Plerixafor (AMD3100) [38], or novel compounds recently described [39].

In this context, regulatory peptide receptors involved in angiogenesis, such as somatostatin receptors (SSTR1-5) overexpressed in several human cancers, might represent another relevant target [40]. Particularly, SSTRs, which mediate the antiproliferative activity of somatostatin in GBM cells [41, 42], can be targeted by specific agonists that, via the activation of specific phosphotyrosine phosphatases [43], exert both cytostatic and antiangiogenic effects in *in vivo* GBM mouse models [44]. Radiolabeled somatostatin analogs are used to localize tumor cells *in vivo,* and radionuclide therapy to treat recurrent GBM is under study [45]. *In vitro* preliminary data indicate that SSTR2 is a suitable target to selectively deliver genes into human GBM cells using viral tools, suggesting that SSTR expression in brain tumors could be exploited for therapeutic approaches. However, to date no clinical data are available using these approaches.

## 3. Advances in Potential Drugs Targeting GBM CSCs

In the course of the last years, several novel compounds and old drugs have been reported to effectively target CSC in different tumor types. Among them, interesting preclinical results are coming out with various biomolecules, such as the natural polyphenol resveratrol [46], the antibacterial and coccidiostatic ionophore salinomycin [47, 48], the new-generation taxoid SBT-1214 [49], and tunicamycin, a N-linked glycosylation (NLG) inhibitor [50], molecules targeting intrinsic signaling pathways of CSCs, such as vismodegib, a hedgehog pathway inhibitor [51], antibodies directed against specific cell surface molecules (including CD44) [52], and, finally, some old drugs, such as metformin used to treat type II diabetes for more than 50 years [53]. Moreover, a high-throughput small molecule screening approach allowed the identification and characterization of chemical compounds potentially effective against GBM CSCs, such as emetine, N-arachidonoyl dopamine, N-oleoyl dopamine (OLDA), and N-palmitoyl dopamine [54].

Below are discussed recent data concerning some compounds that, among others, have been shown to possess selective GBM CSC-inhibitory activity and they appear to be particularly promising for a clinical validation.

### 3.1. Metformin

An old drug that is capturing increasing attention as anticancer agent is the oral antidiabetic drug metformin. This compound, like phenformin and buformin, belongs to the biguanide class. It is modeled after the first isolation of the guanidine derivatives from the French lilac (*Galega officinalis*), a plant known for several centuries to be able to reduce the symptoms of diabetes mellitus. First synthesized in 1929 and tested in the late “50s” on humans as a treatment of diabetes with the trade name “Glucophage” (glucose eater), it was first approved for the treatment of hyperglycemia in the United Kingdom in 1958 and has been widely used in Europe since 1980; several decades later (in 1995) it was also approved by the FDA in the United States. Today it is the most commonly prescribed first line drug for type 2 diabetes [55], and it is also used in polycystic ovarian syndrome, metabolic syndrome, and diabetes prevention [56].

The mechanism of metformin action in diabetes, still not entirely clarified, arises from the suppression of hepatic glucose production and the increase of insulin sensitivity, the reduction of lipolysis in adipocytes, and the reduction of glucose absorption from intestine, resulting in decreased insulin amount and improvement of insulin sensitivity in diabetic patients. These effects are mediated by suppression of mitochondrial respiratory chain, increase of insulin receptor TK activity and stimulation of the GLUT4 transporter to the plasma membrane [57, 58].

Early suggestions of the possible use of metformin in oncology came from epidemiologic studies examining the associations between diabetes, diabetes treatment, and cancer. These studies showed that diabetic patients suffer higher risk of developing cancer than people without diabetes and that adults with diabetes are more likely to die of cancer than their nondiabetic counterparts [59]; nonetheless cancer patients treated with metformin showed considerably reduced tumor burden and incidence [60, 61], cancer-related mortality [62], and cancer risk [63] not only when compared to diabetic patients but in some studies also with respect to nondiabetic subjects [64].

In light of these findings, the potential antitumor effects of metformin have been evaluated in numerous *in vitro* studies on several cancer models including breast [65, 66], endometrial [67, 68], ovarian [69, 70], pancreatic [71, 72], lung [73], prostate [74], head and neck carcinomas [75], acute myeloid leukemia [76], and finally glioma [77].

Moreover, several *in vivo* models have been used to describe the antiproliferative effect of metformin in various tumor types [78–80]. Interestingly, studies have also shown the advantages of combining metformin with standard cytotoxic drugs like cisplatin [81], taxol [82], and doxorubicin [83] or with molecular targeted agents such as gefitinib [84]. It is worth noting that, with regard to GBM, metformin potentiates the proapoptotic effect of TMZ via the modulation of the same intracellular pathway (i.e., activation of 5′-adenosine monophosphate activated protein kinase (AMPK)) [85].

Altogether, these findings strongly highlight the metformin potential as anticancer drug for nondiabetic patients.

The molecular mechanisms of metformin leading to the antiproliferative effects in the tumor models listed above are still currently under intensive investigation. What seems to emerge is that such mechanisms are either indirect, acting on systemic levels of insulin or glucose [86, 87], or direct, having an impact directly on tumor-cell growth and survival.

The main direct mechanism proposed for metformin control of tumor growth inhibition is the activation of AMPK. This enzyme plays a role in cellular energy homeostasis, acting as a metabolic master switch and hence regulating several intracellular systems. Metformin-mediated AMPK activation triggers multiple downstream effects that cooperate to restrain tumor growth. One of the established and most investigated ones is the inhibition of the mTOR pathway [53]. mTOR plays a key role in the control of cell growth, proliferation, and metabolism and mediates the phosphoinositide 3-kinase (PI3K)/Akt signaling pathway, frequently deregulated in human cancers [88, 89] and specifically in CSCs, since it is involved in their survival and maintenance [90].

Other modulators of the cancer inhibitory effects of metformin via AMPK activation include cyclin D1, p21, p27, and p53 [74]. However, emerging evidence showed that metformin may modulate mTOR activity, through AMPK-independent mechanisms [91–93]. Recently, metformin was shown to directly inhibit the enzymatic function of hexokinase I and II in a triple-negative breast cancer model. This action led to cytotoxic effects both *in vitro* and *in vivo*, reducing cancer growth rate under chronic treatment [94].

All these unexpected evidences encouraged the approval of several clinical trials currently ongoing, directed to evaluate the effects of metformin, alone or in combination with standard anticancer drugs, in different neoplastic pathologies (for a complete list refer to [95]).

#### 3.1.1. Metformin and CSCs

A potential novel mechanism of metformin effects emerged in the course of these very recent years: the ability to selectively affect the viability of the CSC subpopulation.

The first report, defining metformin specific action against CSCs by Hirsch et al. [96], demonstrated that breast CSCs, identified in established cell lines and phenotypically characterized by CD44_high_CD24_low_ expression, are sensitive to low doses of metformin without affecting differentiated tumor cell population. Moreover, metformin can deplete CSCs and suppresses breast tumor development when given in combination with doxorubicin [96].

Afterwards, several papers have reported a selective sensitivity to metformin by CSCs in several cancer models. Vazquez-Martin et al. showed that metformin acts synergistically with trastuzumab both *in vivo* and *in vitro* to repress proliferation and survival of CSC in HER2-positive CD44^+^/CD24^−^ breast cancer cell lines [97]. Since metformin can overcome *in vivo* primary resistance to trastuzumab, the authors proposed metformin as promising strategy for treatment of HER2^+^ breast cancer patients [98].

Shank et al. demonstrated the inhibition of ovarian CSC growth and proliferation by metformin, both *in vitro* and *in vivo* [99], associated with a decrease of tumor microvascular density, suggesting that CSC depletion by metformin leads to a reduction in angiogenesis, as reported in different models [81, 100].

Low concentrations of metformin selectively inhibit the proliferation of CD133^+^ pancreatic CSCs, inducing an *in vitro* and *in vivo* anticancer action that reduces cell invasion and tumor formation, effects associated with a reduction of phospho-ERK1/2 and phospho-mTOR accumulation independently of Akt and AMPK activation [101]. Moreover, an *in vitro* and *in vivo* preclinical study reported the specificity of metformin activity towards pancreatic CSCs [102], showing a metformin-mediated increase of mitochondrial production of reactive oxygen species in primary CSC, derived from a set of human pancreatic ductal adenocarcinomas. Finally, metformin showed a synergic effect when used in combination with 5-fluorouracil, in particular affecting CD133+ colorectal cancer cells viability in diabetic patients [103].

Noteworthy, metformin inhibits the inflammatory pathways responsible for CSC formation, probably blocking a metabolic stress response involved in inflammation [104].

The specific effects of metformin against CSCs are promising but need to be extended. Indeed, although a growing number of findings are contributing to deepening of the knowledge of the pathways involved in metformin anticancer action, the detailed mechanisms underlying the eradication of CSCs by metformin are still unclear.

Reduced cancer risk and enhancement of survival associated with metformin exposure reported in population studies fostered speculations about metformin treatment, ascribing beneficial effects to the high concentration directly causing cancer cell death. Moreover, preclinical studies show antineoplastic activity of metformin, but in many cases at concentrations exceeding those achieved in plasma with standard doses used for diabetes [105, 106]. However, this observation did not take into account that chronic treatments, as performed in diabetes or, possibly, in cancer patients, will induce high concentrations in tissues (including brain parenchyma), where metformin preferentially accumulates [107, 108], rather than in plasma.

Furthermore, another important aspect has to be considered to explain the relatively high dose of metformin required to exert antitumor effects *in vitro*, when compared to the dose used in patients with diabetes. *In vitro* tumor cells are generally forced to grow with high concentrations of glucose, serum, or several stimuli such as growing factors, whereas the nutritional conditions of the tumor microenvironment are significantly different. Indeed, glucose concentrations are drastically lower in tumor compared to normal tissues [109]. It has been shown that metformin works synergistically together with 2-deoxyglucose (2-DG, an inhibitor of glucose metabolism) to induce a stronger inhibitory effect on cancer cells viability than the drugs alone [110, 111]. Moreover, Menendez et al. [112] suggested that the low-glucose tumor microenvironment mediates a contextual synthetic lethality that dramatically potentiates the anticancer effect of metformin.

However, beside the relevance of metformin doses in experimental model and clinical settings, it is conceivable that translational studies should evaluate metformin at conventional antidiabetic doses but also investigate more aggressive dosing to recognize distinct mechanisms of action relevant for antineoplastic activity.

#### 3.1.2. Metformin and GBM CSCs

Recently, we and others have shown that metformin effectively affects GBM CSCs proliferation and survival [113, 114].

**Figure 1:**
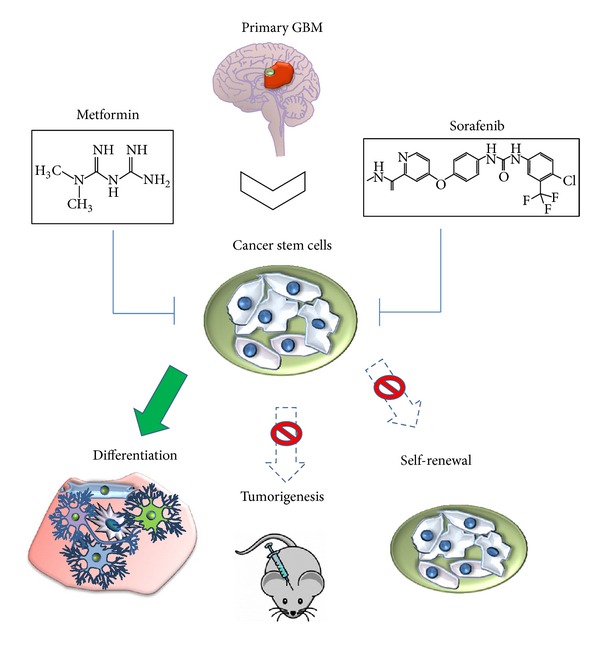
Cellular mechanisms of metformin and sorafenib effects against GBM CSCs. Metformin and sorafenib selectively target CSCs from which GBMs develop, acting on three key features: self-renewal, tumorigenicity, and differentiation ability. Fading arrows mean that metformin and sorafenib restrain specific CSC features, while the green arrow depicts an induction towards CSC differentiation mediated by metformin and sorafenib.

Noteworthy, in both studies, experimental data are built on a similar model based on CSC enriched cultures derived from postsurgical samples of human GBMs [115] (Figure 1). In particular, we showed that metformin powerfully inhibits CSC viability, with significantly higher efficiency than what occurred in differentiated GBM cells or normal stem cells (umbilical cord mesenchymal stem cells, MSCs). Moreover, metformin impairs *in vitro* GBM CSC self-renewal, as measured by spherogenic activity. Different intracellular transduction mechanisms, such as Akt and mTOR pathways, seem to be modulated in an inhibitory way in CSCs but not in differentiated GBM cells or in MSCs, after metformin treatments, but the detailed mechanisms of these differences are still to be clarified.

Interestingly, within CSC subpopulations, cells expressing CD133 showed higher sensitivity to the antiproliferative effects of metformin than nonselected (overall) GBM CSCs. Similar data have been recently reported in pancreatic adenocarcinoma cells [101]. Since CD133 expression seems to be related to chemoresistance [116], the ability of metformin to preferentially target CD133^+^ cells highlights the possibility that this drug could overcome pharmacological resistance in GBM or in other sensitive tumors.

Metformin was also described to be an activator of the transcription factor FOXO3 that via AMPK-dependent mechanisms leads to differentiation of GBM CSCs, accompanied by loss of tumor-initiating potential. Moreover, the suppression of GBM CSCs-tumor formation implanted in the brain parenchyma and a significant extended mouse survival were showed after systemic administration of metformin [113].

Along with the fact that metformin has already been safely used in the clinic and that it penetrates the blood-brain barrier [107, 117], these findings suggest that metformin could be a candidate for GBM clinical treatment. However, further preclinical and clinical studies are required to confirm these experimental evidences.

Regarding the use of metformin in GBM patients, a phase I clinical trial is currently ongoing (clinicaltrials.gov NCT01430351) aimed to find the highest tolerable dose of TMZ in combination with memantine (a NMDA receptor antagonist), mefloquine (an antimalarial drug), and/or metformin, which can be given to patients with GBM who have already been subjected to radiation and chemotherapy.

### 3.2. Sorafenib in GBM and CSCs

Sorafenib (SO) is an oral multikinase inhibitor, which targets several tyrosine kinases receptors (RTK), such as VEGFR2 and VEGFR3, PDGFR*β*, fibroblast growth factor receptor 1 (FGFR1), and Flt-3, RET, and c-Kit, all involved in tumor growth progression and neoangiogenesis. Furthermore, SO directly inhibits the downstream serine/threonine kinase Raf (Raf1 and both wt and mutated B-Raf), a key member of the MEK/ERK signal transduction pathway.

Both Raf/MEK/ERK-dependent and -independent mechanisms have a role in the antitumor effects of SO. However, the defined molecular mechanisms have not been clarified yet [118].

SO appears to be a promising anticancer agent: it was shown to be effective in numerous clinical studies with several standard chemotherapeutic drugs such as doxorubicin [119], and it has been already approved by FDA and EMA for the treatment of patients with advanced renal-cell carcinoma [120] and hepatocellular carcinoma [121].

Concerning GBM, SO possesses *in vitro* and *in vivo* antitumor activity in glioma cell lines or primary cultures [122, 123].

It was recently shown that SO inhibited the proliferation of human GBM CSCs, through inhibition of MAPK and PI3K/Akt. Interestingly, SO determined the downregulation of the antiapoptotic member of Bcl-2 family, Mcl-1, an effect that was determinant for the induction of GBM TICs apoptosis [124]. Moreover, *in vitro* (clonogenic ability reduction) and *in vivo* (tumorigenic potential inhibition, reducing the source of feeding of the tumor mass) results lead to the conclusion that SO preferentially affects TIC subpopulation (Figure 1).

SO selective effect on GBM CSCs is corroborated by the lower effectiveness of SO on GBM differentiated cells compared with the same cultures maintained in “stem-permissive” conditions. Interestingly, after SO treatment, survived cell population showed enrichment in GFAP- and MAP2-expressing cells, with concomitant reduction of subpopulations expressing Sox2, nestin, and Olig2, three crucial CSC markers [124]. All these effects were also associated with a significant reduction of tumorigenicity, clearly indicating a selective depletion of TICs in the cultures after SO treatment (Figure 1).

Further *in vitro* and *in vivo* studies are required to confirm these data and to deeper investigate the mechanism of SO action.

Clinical trials aimed to verify safety, tolerability, and efficacy of SO in combination with TMZ, bevacizumab, or radiotherapy in newly diagnosed or recurrent GBM patients are ongoing (http://www.clinicaltrials.gov). Although SO could be safely administered with TMZ, limited activity in a phase II trial in patients with recurrent disease was reported [125].

Additionally, SO did not improve the efficacy of treatment in comparison with TMZ alone [126]; however, more than 40% of patients did not receive any maintenance SO because of disease progression.

Nonetheless, a recent phase I trial demonstrated that SO can be safely combined with radiation and TMZ in patients with high-grade glioma and with radiation alone in patients with recurrent glioma [127]. Moreover, a new phase II study showed that the combination of SO and TMZ was feasible and safe, with a partial activity in patients with relapsed GBM [128].

Further investigations are required to have more detailed therapeutic indications.

### 3.3. Disulfiram

Disulfiram (tetraethylthiuram disulfide, DSF) is an inhibitor of the aldehyde dehydrogenase (ALDH) enzyme family, widely and safely used for alcoholism treatment. DSF is able to reduce *in vitro* cell growth and self-renewal of TMZ-resistant GBM stem cells. This effect seems mediated by inhibition of Polo-like kinase 1, a brain cancer overexpressed serine/threonine kinase, involved in cell cycle regulation [129, 130]. Moreover, DSF abolishes stem-like cell population in GBM cell lines likely activating apoptotic pathway via modulation of the Bcl-2 family and enhances the cytotoxic effects of gemcitabine [131].

Additional evidence of the selective effect of DSF towards CSCs has been recently reported in an *in vitro* model of triple-negative breast cancer [132]. This experimental evidence should be extended and strengthened by further *in vitro* and *in vivo* preclinical studies to admit DSF clinical trials either as monotherapy or adjuvant with other agents. Indeed, DSF, which penetrates the blood-brain barrier, is an attractive drug because of its safety, as shown by its use for the treatment of alcohol abuse for decades [133, 134].

## 4. Conclusions

Several studies provide supporting evidence of the existence of CSCs in GBM, and much of the ongoing neurooncology research focuses on better understanding the definite role of CSCs in GBM pathogenesis, recurrence, and therapy. CSC hypothesis well explains GBM heterogeneity and resistance of these tumors to conventional therapies and gives a boost to identify novel drugs and reconsider old molecules. As CSCs have distinctive properties from cells forming the bulk of the tumor, thus innovative experimental and pharmacological approaches have been refined to preferentially target and eradicate these residual chemo- and radioresistant cells able to regenerate GBM. Perhaps, future significant improvement in the targeting of the CSC subpopulation, possibly providing synergistic effect with conventional treatments, may increase the efficacy of GBM therapy.
